# SARS-CoV-2 Lineage A.27: New Data from African Countries and Dynamics in the Context of the COVID-19 Pandemic

**DOI:** 10.3390/v14051007

**Published:** 2022-05-09

**Authors:** Anissa Chouikha, Adamou Lagare, Kais Ghedira, Amadou Diallo, Richard Njouom, Safietou Sankhe, Fawzi Derrar, Kathleen Victoir, Koussay Dellagi, Henda Triki, Moussa Moise Diagne

**Affiliations:** 1 Laboratory of Clinical Virology, WHO Reference Laboratory for Poliomyelitis and Measles in the Eastern Mediterranean Region, Pasteur Institute of Tunis, University Tunis El Manar (UTM), Tunis 1002, Tunisia; henda.triki@pasteur.tn; 2 Reasearch Laboratory “Virus, Vectors and Hosts: One Health Approach and Technological Innovation for a Better Health”, LR20IPT02, Pasteur Institute, Tunis 1002, Tunisia; 3 Centre de Recherche Médicale et Sanitaire (CERMES), BP: 10887—634 Bd de la Nation, Niamey YN034, Niger; lagare@cermes.org; 4 Laboratory of Bioinformatics, Biomathematics and Biostatistics (BIMS), Institut Pasteur de Tunis (IPT), 13, Place Pasteur BP 74, University of Tunis-El Manar, Tunis 1002, Tunisia; ghedirakais@gmail.com; 5 Institut Pasteur Dakar, 36 Avenue Pasteur, Dakar BP 220, Senegal; amadou.diallo@pasteur.sn (A.D.); safietou.sankhe@pasteur.sn (S.S.); moussamoise.diagne@pasteur.sn (M.M.D.); 6 Centre Pasteur of Cameroon, Centre Pasteur of Cameroon, 451 rue 2005, Yaoundé, Cameroon; njouom@pasteur-yaounde.org; 7 Institut Pasteur d’Algérie, 01 Rue du Petite Staouéli, Dély-Brahim, Algiers 16047, Algeria; fawziderrar@gmail.com; 8 Institut Pasteur, Direction Internationale, Pôle Coopération Scientifique, 25 Rue du Dr Roux, 75015 Paris, France; kathleen.victoir@pasteur.fr; 9 Pasteur Network Association, Institut Pasteur 25 Rue du Dr Roux, 75015 Paris, France; koussay.dellagi@pasteur.fr

**Keywords:** SARS-CoV-2, lineage A.27, tMRCA, phylogeography, phylogeny, clade 19B

## Abstract

SARS-CoV-2 is constantly evolving with lineages emerging and others eclipsing. Some lineages have an important epidemiological impact and are known as variants of interest (VOIs), variants under monitoring (VUMs) or variants of concern (VOCs). Lineage A.27 was first defined as a VUM since it holds mutations of concern. Here, we report additional lineage A.27 data and sequences from five African countries and describe the molecular characteristics, and the genetic history of this lineage worldwide. Based on the new sequences investigated, the most recent ancestor (tMRCA) of lineage A.27 was estimated to be from April 2020 from Niger. It then spread to Europe and other parts of the world with a peak observed between February and April 2021. The detection rate of A.27 then decreased with only a few cases reported during summer 2021. The phylogenetic analysis revealed many sub-lineages. Among them, one was defined by the substitution Q677H in the spike (S) gene, one was defined by the substitution D358N in the nucleoprotein (N) gene and one was defined by the substitution A2143V in the ORF1b gene. This work highlights the importance of molecular characterization and the timely submission of sequences to correctly describe the circulation of particular strains in order to be proactive in monitoring the pandemic.

## 1. Introduction

Since its onset, the SARS-CoV-2 pandemic has been characterized by the emergence of several viral lineages. Some of them rapidly spread over the world due to their increased transmissibility and displaced older lineages. Others were associated with increased virulence and/or their ability to escape the host immune responses. Tracking the circulation of variants and lineages is, currently, one of the most important pillars of SARS-CoV-2 surveillance worldwide. As of November 2021, the WHO (World Health Organization) had identified four variants of concern (VOCs): B.1.1.7 (Alpha) which originated from the United Kingdom [1], B.1.351 (Beta) from South Africa, P.1 (Gamma) from Brazil and B.1.617.2 (Delta) from India [2]. At that time, the Delta variant displaced all the other variants [3,4] and was subdivided into sub-lineages from AY.1 to AY.133 in the Pango lineage designation system [5]. Recently, in November 2021, a new VOC, B.1.1.529 (Omicron), was described in Botswana and South Africa, which has rapidly spread all over the world [6]. These VOCs have shown sustained regional community transmission followed by quick worldwide dispersal. The Alpha variant was the first detected with an international spread; it was displaced by the Delta variant, and presently, Omicron is the most detected variant all over the world [2].

In addition, other variants presenting mutations potentially associated with altered phenotypes are labeled as variants of interest (VOIs) or variants under monitoring (VUMs) according to the WHO classification, or variants under investigation (VUIs) according to the European Centre for Disease Prevention and Control (eCDC) or Public Health England (COG-UK) classifications [2,7,8]. These variants are subject to attentive surveillance, to better understand how easily they might be transmitted and the effectiveness of currently authorized vaccines against them. The A.27 lineage was first classified as a VUM by the eCDC and the COG-UK [7,8] and as a VOI by the French Society of Microbiology [9] since it shows a mutational pattern similar to VOCs and VOIs, with several amino acid substitutions along the viral genome, especially in the S gene [10,11,12,13]. Among these amino acid substitutions, L18F, L452R and N501Y are located in the N-terminal (NTD) or receptor binding domain (RBD) regions and have been suggested to result in immune escape and higher transmissibility [14,15,16,17]. In contrast, A.27 does not have the D614G mutation, typical of the SARS-CoV-2 B lineage and which is now predominant worldwide. At the time of writing, the A.27 lineage was first detected in Denmark in mid-December 2020 and then reported in 38 countries: 17 from Europe, and 16 from Africa, together with the USA, India, Indonesia, Iceland and Australia. However, almost half of the sequences reported in the GISAID database are from France [18].

The present work, implemented in the frame of a collaborative monitoring conducted by a consortium of 10 African institutes of the Pasteur Network (REPAIR consortium), reports additional lineage A.27 sequence data from 5 countries in Africa (Niger, Tunisia, Senegal, Algeria and Cameroon), describes the molecular characteristics of these sequences and their classifications in comparison with other A.27 public available data and traces the dynamic evolutionary history of this lineage worldwide.

## 2. Materials and Methods

A total of 85 new SARS-CoV-2 sequences belonging to lineage A.27 were identified and included in the present study; they originated as follows: 46 from Niger, 23 from Tunisia, 14 from Senegal, 1 from Cameroon and 1 from Algeria (Table 1).

### 2.1. NGS Sequencing

Whole genome sequencing was performed for the 85 samples using Illumina or Nanopore (MinION) technologies. Viral RNA was extracted from a 140 μL nasopharyngeal sample with the Qiamp viral RNA mini kit (Qiagen, Hilden, Germany) according to the manufacturer’s instructions. SARS-CoV-2 detection was performed using real-time PCR corresponding to the SARS-CoV-2 detection kit (The LightMix^®^ E-gene kit (TIB Molbiol, Berlin, Germany)). SARS-CoV-2-positive samples with a Ct value < 30 were selected for NGS sequencing. Genomes were extracted by an amplicon-based approach using the United States Food and Drug Administration (US FDA)-approved Illumina COVIDSeq kit [19], a modified version of the ARTIC protocol. The extracted RNAs were reverse transcribed to single-strand cDNA as per the manufacturer’s instructions, and SARS-CoV-2 PCR was performed with two specific primer pools combined with proven Illumina sequencing technology, which allowed obtaining 98 tiled 400 bp amplicons covering the whole genome. For each sample, PCR products were combined, and libraries were prepared using the Illumina Nextera DNA UD Indexes as per the manufacturer’s instructions. Libraries were purified with AMPure XP magnetic beads (Beckman Coulter, Brea, CA, USA), and the concentration was measured using a Qubit dsDNA HS Assay kit (Thermo Fisher Scientific, Waltham, MA, USA). Library pool validation and mean fragment size were determined using a Bioanalyzer 2100 (Agilent, Santa Clara, CA, USA) as per the manufacturer’s instructions. The 400 bp library pool was diluted to 4 nM. Libraries were pooled, denatured and diluted to 1.4 pM and then sequenced on a NextSeq550 instrument with a V2.5 High Output Cartridge 2 × 150 bp run kit (Illumina, San Diego, CA, USA). Sequences’ raw data were processed using fastqc version 0.11.9 for quality control (https://www.bioinformatics.babraham.ac.uk/projects/fastqc/) (accessed on 12 November 2021). Low-quality reads and adapters were filtered using trimmomatic version 0.39 with a Phred quality score of 30 as the threshold. Filtered reads were mapped against the SARS-CoV-2 reference (NC_045512) using the Burrows–Wheeler Aligner MEM algorithm (BWA-MEM) (v0.7.7). SAM tools were used to sort BAM files, to generate alignment statistics and to obtain coverage data [20]. The obtained whole genome sequences are listed in Table 1.

### 2.2. Genome Assembly

The genomes were assembled using the EDGE COVID-19 pipeline which is based on the fully open source EDGE Bioinformatics software [21]. FaQCs was used to control the quality of the reads [22]. Low-quality regions of reads were trimmed and filtered if the reads failed a quality threshold of 20 or minimum length of 50bp. The reads that passed the QC process were then aligned with the original Wuhan-Hu-1 complete reference genome (RefSeq accession: NC_045512.2) using BWA-MEM [23]. Various parameters were defaulted: (i) minimum depth coverage (5x) to support a variant site, (ii) alternate base threshold (0.5) to support an alternative for the consensus base to be changed, (iii) indel threshold (0.5) to support an INDEL for the consensus base to be changed and (iv) minimum mapping quality [24].

### 2.3. Sequences Retrieved from GISAID Database

A total of 597 SARS-CoV-2 whole genome sequences belonging to the A.27 lineage originating from 34 countries and available in GISAID at the time of writing were retrieved. The number of sequences per country was as follows: Australia (*n* = 1), Austria (*n* = 7), Belgium (*n* = 21), Benin (*n* = 21), Burkina Faso (*n* = 15), Cote d’Ivoire (*n* = 25), Denmark (*n* = 2), England (*n* = 16), France (*n* = 276), Gabon (*n* = 1), Germany (*n* = 34), Greece (*n* = 1), Guinea (*n* = 1), Iceland (*n* = 1), India (*n* = 3), Indonesia (*n* = 1), Ireland (*n* = 2), Italy (*n* = 9), Luxembourg (*n* = 4), Mayotte (*n* = 13), the Netherlands (*n* = 5), Nigeria (*n* = 1), Rwanda (*n* = 1), Scotland (*n* = 1), Senegal (*n* = 3), Slovenia (*n* = 41), Spain (*n* = 10), Sudan (*n* = 1), Sweden (*n* = 13), Switzerland (*n* = 23), Togo (*n* = 23), Tunisia (*n* = 1), Turkey (*n* = 9) and the USA (*n* = 8).

### 2.4. Phylogenetic Analysis

Phylogenetic analysis was performed on 280 whole genomes (among the 597 retrieved from GISAID and those generated in the frame of the REPAIR consortium). This subset includes sequences from 35 different countries. All sequences with more than 15% of ambiguous nucleotides (Ns) throughout the whole genome were not included. Additionally, for countries with a large number of sequences such as France, only a subset of sequences selected randomly was included. Sequence alignment was performed with MAFFT using default parameters [25]. The resulting alignment was used to build a maximum likelihood phylogenetic tree using the IQ-TREE web server, supported by 1000 bootstrap replicates (http://iqtree.cibiv.univie.ac.at/) (accessed on 12 November 2021). The phylogenetic tree was then visualized using Figtree software (http://tree.bio.ed.ac.uk/software/figtree/) (accessed on 12 November 2021). The tree was rooted using the midpoint rooting method. The number of sequences per country was as follows: Algeria (*n* = 1), Australia (*n* = 2), Austria (*n* = 3), Belgium (*n* = 7), Benin (*n* = 11), Burkina Faso (*n* = 3), Cote d’Ivoire (*n* = 7), Denmark (*n* = 1), England (*n* = 13), France (*n* = 90), Gabon (*n* = 1), Germany (*n* = 9), Ghana (*n* = 1), Greece (*n* = 1), Guinea (*n* = 1), India (*n* = 2), Indonesia (*n* = 1), Ireland (*n* = 2), Italy (*n* = 5), Luxembourg (*n* = 2), Mayotte (*n* = 1), the Netherlands (*n* = 6), Niger (*n* = 30), Nigeria (*n* = 1), Rwanda (*n* = 1), Scotland (*n* = 1), Senegal (*n* = 11), Slovenia (*n* = 19), Spain (*n* = 5), Sweden (*n* = 2), Switzerland (*n* = 14), Togo (*n* = 7), Tunisia (*n* = 8), Turkey (*n* = 8) and the USA (*n* = 4).

### 2.5. The Phylogeography Construction

Phylogeography analysis was based on 330 S gene sequences among the 597 whole genome sequences; only those with zero ambiguous nucleotides in the S gene were included. They were collected from 27 countries as follows: Australia (*n* = 1), Austria (*n* = 2), Belgium (*n* = 10), Denmark (*n* = 2), England (*n* = 13), France (*n* = 157), Germany (*n* = 17), Greece (*n* = 1), Ireland (*n* = 1), Italy (*n* = 8), Luxembourg (*n* = 4), Mayotte (*n* = 10), the Netherlands (*n* = 6), Nigeria (*n* = 1), Niger (*n* = 21), Rwanda (*n* = 1), Scotland (*n* = 1), Senegal (*n* = 4), Slovenia (*n* = 22), Spain (*n* = 4), Sweden (*n* = 2), Switzerland (*n* = 16),Togo (*n* = 8), Tunisia (*n* = 6), Turkey (*n* = 8) and the USA (*n* = 4).

The epidemic history of the A.27 lineage was investigated with a coalescent-based strategy inferred by Bayesian Markov chain Monte Carlo (MCMC) analysis implemented in BEAST software version 1.8.4 (http://beast.bio.ed.ac.uk) (accessed on 12 November 2021). For this analysis, a parametric model of coalescent population growth corresponding to the coalescent Bayesian Skygrid under the uncorrelated lognormal relaxed clock was used, allowing the evolutionary rate to vary among taxa. To find the best substitution model to use with BEAST, “Find Best DNA/Protein Models (ML)” in MEGAX was used. The HKY nucleotide substitution model with gamma-distributed rates among sites was identified as one of the best models based on Bayesian information criterion (BIC) and Akaike information criterion (AIC) criteria and was used under the relaxed molecular clocks. The gamma distribution allows for several gamma rate categories to be applied to the substitution rate, allowing gamma rate heterogeneity. MCMC was run using default parameters, with a chain length of 100,000,000 generations for the SARS-CoV-2 A.27 sequences in order to achieve an effective sample size (ESS) ≥ 200, sampled every 100 thousand steps with 10% of the initial runs excluded. The BEAST output log file was analyzed with the TRACER v1.6 program (available from http://tree.bio.ed.ac.uk/software/tracer/) (accessed on 12 November 2021). A tree was generated using the MCMC algorithm, being part of the BEAST package (version 1.8.4). The TreeAnnotator program was used to summarize and construct the maximum clade credibility tree (MCC) from 1000 trees. Briefly, the burnin (as states) was fixed to 100, equivalent to 10% of the total number of trees generated by BEAST. The posterior probability limit was fixed at 0.7. Posterior summaries were only calculated for nodes in the target tree that have a posterior probability greater than the specified limit. Nodes with very little support were not considered.

Phylogeographic analysis was performed using the BEAST package to estimate the diffusion process of the SARS-CoV-2 A.27 lineage in the world, and particularly in Africa. The capital of each country was used as a discrete location state, and the sampling date was used to calibrate the time scale. The MCC tree was uploaded into SPREAD to generate a keyhole markup language file (kmL) (Supplementary Material S1). The selected sequences were assigned to a total of 27 distinct geographic groups corresponding to each country. In order to provide a spatial projection, the migration routes indicated by the tree were visualized using Google Earth Pro version 7.3.4 to identify significant migrations.

## 3. Results

A total of 682 sequences belonging to the A.27 lineage were included in the present study: 85 are from Niger, Tunisia, Senegal, Cameroon and Algeria, and 597 are from other countries and were retrieved from the GISAID database as of 12 November 2021.

**Figure 1:**
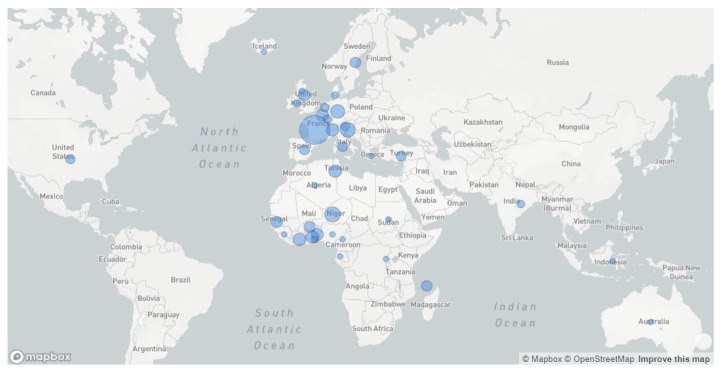
Geographic distribution of SARS-CoV-2 lineage A.27 from November 2020 to November 2021 based on 682 sequences from 38 different countries.

The demographic and clinical data related to the 85 A.27 samples reported as part of the present work are shown in Table 1. The sequences were isolated from 60 males and 25 females. Out of the 60 patients for whom the clinical form was provided, 35 were asymptomatic (58.3%), 23 presented with mild infection (38.3%) and only 2 (3.3%) had a severe infection (Table 1). The age ranged from less than 1 to 85 years (mean age = 41.5 ± 19.2 years). Most cases were due to an apparent indigenous transmission except for two travelers who came to Tunisia and were detected positive upon arrival (one was arriving from Libya and the other from Côte d’Ivoire). In contrast, most of the cases reported from Senegal were detected in Chinese outgoing travelers who seemingly were infected in Senegal. As of November 2021, the A.27 variant was reported in 38 different countries from the 5 different continents: most of them were in Europe (*n* = 18 countries), and from Africa (*n* = 16 countries); the variant was also reported in the USA, India, Indonesia and Australia. The worldwide geographic distribution of the A.27 sequences including those previously reported in GISAID and the ones reported for the first time in the present work is shown in Figure 1.

The earliest isolates of A.27 were detected in November 2020 (weeks 48 and 49) and were from Niger, reported here for the first time. The number of reported sequences increased up to February/March 2021 (weeks 5-2021 to 9-2021) and then decreased. The last reported sequences at the time of writing were from Turkey, with a collection date of 5 August 2021.

**Figure 2:**
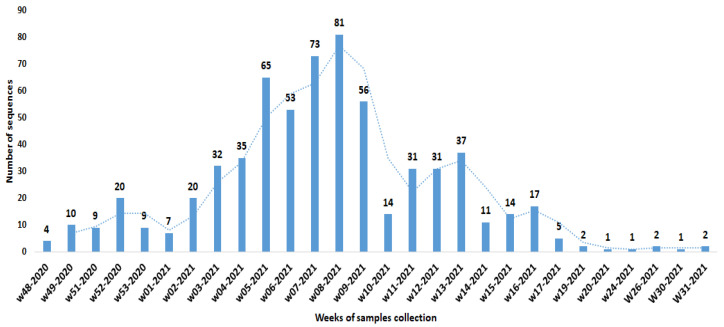
Timeline of SARS-CoV-2 lineage A.27 detection worldwide per epidemiological week based on 643 sequences as of 12 November 2021.

Figure 2 shows the number of reported sequences according to their date of isolation. The histogram is based on 643 out of the 682 A.27 sequences, reported in GISAID and in the present work (the remaining sequences were published with no or incomplete date of isolation and could not be included in the graph).

Among the sequences retrieved from GISAID and those reported in this work, 280 had good coverage through the whole genome and were selected with a maximum of 15% of ambiguous nucleotides through the whole genome to study the mutational profile and the phylogenetic grouping of the A.27 lineage. Based on these sequences, Supplementary Material S2 shows the main amino acid (aa) substitutions that characterize the A.27 lineage. Fourteen aa substitutions are found in almost all the reported sequences (more than 96%): P286L, D2980G and N3651S in ORF1a, P1000L in ORF1b, L18F, L452R, N501Y, A653V, H655Y, D796Y and G1219V in the S protein, V50A in ORF3a, L84S in ORF8 and S202N in the N gene. In addition to the aa substitutions, two deletions, one at position 257/258 in ORF3a and one at position 119/120 in ORF8, are present in 99.6% (*n* = 279) and 99.3% (*n* = 278) of the sequences, respectively.

**Figure 3:**
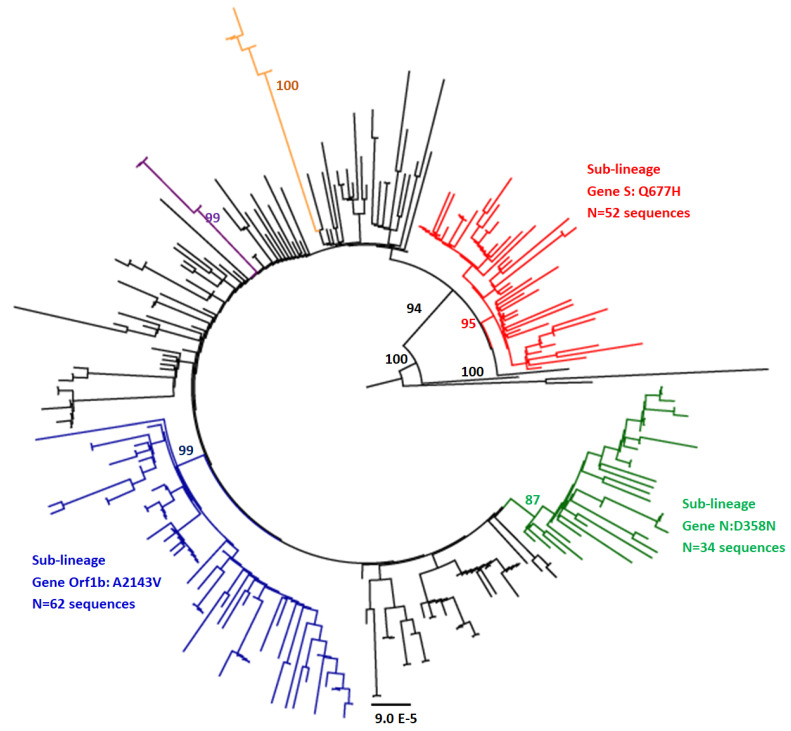
Phylogenetic tree of 280 whole genomes of SARS-CoV-2 lineage A.27. The tree was constructed using the maximum likelihood method using the IQ tree web server and visualized by FigTree. Topology was supported by 1000 bootstrap replicates.

Nineteen other aa substitutions are less frequently found. The three most frequent ones are A2143V in the ORF1b gene (exactly in NSP15), D358N in the N gene (*n* = 34, 12.1%) and Q677H in the S protein detected in (*n* = 62; 22.1%) and (*n* = 52, 18.2%) of the sequences (Supplementary Material S1). Phylogenetic analyses, performed on the 280 complete genome sequences (Figure 3), revealed the presence of at least four genetic groups that are not generally time- or country-specific. Group1 (in red) includes 52 sequences isolated between November 2020 and April 2021 from 15 different countries, where all of them hold the Q677H minor aa substitution in the S gene. Group2 (in blue) includes 62 sequences from 8 different countries, isolated from November 2020 to April 2021, where all of them hold the A2143V minor aa substitution in ORF1b. Group3 (in green) includes 34 sequences isolated between December 2020 and April 2021 from 12 different countries, where all of them hold the substitution D358N in the N gene. The latest sequences reported from Turkey, isolated from June to August 2021, and those reported from Italy showed a different profile of minor mutations and are represented in the phylogenetic tree in purple and orange, respectively (Figure 3).

The sequences from Turkey hold minor aa mutations, namely, A2460V, L3495F and S3675F in the orf1a gene, L1701F in the ORF1b gene and T223I in the ORF3a gene, and the Italian sequence hold the mutations T95I, T572S and H1083Y in the S gene.

For the phylogeographic analysis, 330 sequences corresponding to the S gene and belonging to A.27 genomes were investigated in the present study; a total of 300 were retrieved from the GISAID database as of 24 August 2021, and 30 with zero ambiguous nucleotides in the S gene are part of the 85 WGSs reported for the first time in the present study.

### 3.1. Most Recent Common Ancestor (tMRCA)

The tMRCA of the progenitor virus that ultimately gave rise to all SARS-CoV-2 A.27 strains circulating in the world was estimated around 20 April 2020 with an HPD (highest posterior density) of 95% (January 2020, August 2020).

### 3.2. Phylogeography Analysis

**Figure 4:**
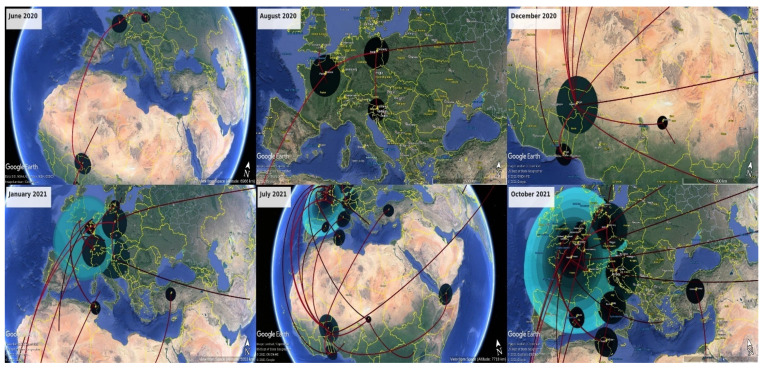
Phylogeographic analysis of SARS-CoV-2 lineage A.27 based on 330 S genes from 27 countries.

To gain insight into the spatial temporal dynamics of the diffusion process of the SARS-CoV-2 A.27 variant in Africa and in the world, the spatial estimates annotated in the S gene region MCC tree were mapped on Google Earth Pro version 7.3.4 (http://www.google.com/earth/download/ge/) (accessed on 12 November 2021). This mapping allows one to visualize the virus’s geographic spread process over time. The links between different geographic regions represent branches in the MCC tree on which virus migration occurs, and the circle areas reflect the number of branches maintaining a particular location at that time point (Supplementary Material S1). Figure 4 shows the temporal dynamics of A.27’s spatial dispersal processes in the world.

Based on the investigated A.27 S gene sequences, the phylodynamic and phylogeographic analysis revealed that the virus started its spread from Niger in Africa. The virus faced a big spread in March and April 2020 and then migrated from Niger to France starting from April 2020. Once in France, the virus migrated rapidly to Germany in the same month when the virus started to spread in Europe. At the same time, the virus migrated from Niger to reach Togo in June 2020. In parallel, the A.27 lineage continued its spread in Niger in July and August 2020. From August 2020, the A.27 lineage saw a big spread in Africa and Europe. Indeed, starting from July 2020, the virus migrated from Germany to reach Austria in August 2020, and from France to the United Kingdom and Belgium in September 2020, to Luxembourg in December 2020 and to Senegal in early 2021. The virus migrated from Niger to Mayotte around August 2020 and to Senegal in December 2020, and then to Turkey around January 2021. The virus seemed to migrate from Senegal to reach Rwanda in April 2021. The virus migrated from the United Kingdom around September 2020 to reach Tunisia where it spread in December 2020. The A.27 variant spread worldwide in 2021, with two distinct introductions in the USA from Niger around February 2021 and in France by April 2021, as well as migration from Germany to reach Australia by March 2021.

## 4. Discussion

A.27 is a SARS-CoV-2 variant that was reported in late 2020 [7,8,9,10,11,12]. Its genome holds several mutations along the viral genome, some of which are shared with other VOCs and VOIs [7,8]. The present study on the COVID-19 pandemic in Africa was conducted in the frame of a collaborative investigation led by the Pasteur Network, the REPAIR program, which is a multidisciplinary exploration in the context of the pandemic to determine, among others, the molecular epidemiology of SARS-CoV-2 in Africa.

We reported 85 new sequences from African countries and provided an overview of the A.27 lineage genetic characteristics and its epidemiology in Africa and worldwide, based on the new and previously reported sequences in the GISAID database.

Up to November 2021, sequences belonging to the A.27 lineage were reported from 38 different countries, most of them from Europe (N = 17) and Africa (*n* = 16). At the time of writing, and based on the sequences submitted in the GISAID database [18,26], the first reported sequences were from Denmark, dated to 14 December 2020, but we reported sequences from Niger, detected as early as November 2020. This lineage was described as a variant holding the N501Y substitution in the S gene, which increases the transmissibility of the virus [13]; also note that the A.27 lineage has a G in amino acid residue 614 of the S protein, while most of the circulating lineages have the D614G change [26]. This lineage was then described in Mayotte which is a French territory [27]. Then, after, lineage A.27 was named the Henry Mondor variant since it caused many grouped cases in France [11]. Indeed, the highest number of sequences was reported in France (*n* = 276), and most of the cases were reported in February–March 2021; then, the frequency of detection of the variant declined [11]. The same situation was found in Germany where the first appearance of the lineage dated to early January; the frequency of A.27 then increased until the end of February and then declined concomitantly with the increase in the B.1.1.7 UK lineage [10,28]. This lineage was also described in Cote d’Ivoire in January–February 2021 [11]. In our series, most of the A.27 sequences from Tunisia were detected in February and April 2021, and the lineage was then apparently displaced by the B.1.1.7 UK lineage that was introduced in February 2021 [29]. Most sequences from Niger were detected earlier, in November–December 2020, with only few sequences in January and February 2021. The indigenous sequences from Senegal dated to December 2020 to February 2021. The other sequences reported in this work were from outgoing Chinese travelers living in the same environment/district in Dakar city. Interestingly, some of these travelers were returning from a stay in China less than a week before the detection of the infection. This is even more interesting given that, to our knowledge, no A.27 strain has been identified in China thus far. In addition, although it seems that the detection of the A.27 lineage is becoming less frequent worldwide, it is noteworthy that new sequences from Turkey (July–August 2021) were recently reported, indicating recent active circulation, and they should be further monitored.

We also studied the mutational profile and the phylogenetic grouping of the A.27 lineage based on the sequences reported in GISAID since the emergence of the lineage up to date. Derived from the 19B clade and with a D614 background, fourteen aa substitutions are found in almost all the A.27 sequences and can be considered as the A.27 lineage-defining mutations, namely, P286L, D2980G and N3651S in ORF1a, P1000L in ORF1b, L18F, L452R, N501Y, A653V, H655Y, D796Y and G1219V in the S protein, V50A in ORF3a, L84S in ORF8 and S202N in the N gene. Among the seven mutations in the S gene, three are also known from other VOCs and VOIs (L18F, L452R and N501Y) located in the NTD or RBD epitope regions of the S protein and were previously reported to result in immune escape and/or higher infectivity and transmissibility [14,15,16,17,30,31]. The mutations A653V and H655Y, also in the RBD, are in proximity to the S1/S2 furin cleavage site, at position 681 that promotes infection and cell–cell fusion [32]. D795Y is close to the essential TMPRSS2 cleavage site [33] at position 815. The L18F replacement in the S protein is part of an antigenic supersite in the N-terminal domain of the S protein and also appears in VOCs that are associated with immune escape and reinfection, such as B.1.351 and P.1. However, data from the literature and from GISAID do not reveal a particular severity of this variant, which is also confirmed in our series. More than 90% of the sequences that we reported in this work are from asymptomatic patients or mild clinical forms.

In addition to these lineage-defining aa substitutions, other less frequent mutations are also found; some of them define special sub-clusters in the phylogenetic tree of A.27 sequences and are supported by high bootstrap values. Indeed, two major sub-clusters within the A.27 lineage are found: one includes 52 sequences holding the Q677H substitution in the S gene (Figure 4, cluster in red), and the other includes 62 sequences all having the A2143V substitution in ORF1b (Figure 4, cluster in green). The substitution Q677H in the S gene was previously described in other SARS-CoV-2 lineages from clades 20G, 20A and 20B. Located near the furin binding pocket, it was suggested that it might be of importance to S1/S2 cleavage [34]. Q677H and A2143V together with several minor mutations were mainly found up to April 2021, the period where A.27 was actively circulating. The few sequences detected from May to August 2021, including the most recent sequences in Turkey, have different mutational profiles in addition to the fourteen lineage-defining main mutations.

Lineage A.27 belongs to clade 19B which spread from the beginning of the pandemic as well as clade 19A; this lineage was overtaken by the viruses of clades 20A, 20B and 20C which carry the D614G mutation in the S gene [10]. SARS-CoV-2 viruses of clade 19B were rarely detected after the first wave of the COVID-19 pandemic and practically disappeared until its re-emergence in the last quarter of 2020, where several teams have observed the re-emergence of different variants of clade 19B, especially with the emergence of lineage A.27 [9,10,11,26]. The phylodynamic analysis showed a high spread of the lineage starting from August 2020; this date was estimated by Calvignac-Spencer et al. as the most common ancestor’s emergence date based on the sequences reported as of May 2021 [9]. The tMRCA was also estimated to have emerged in late September by the team of Kaleta et al. based on samples retrieved from GISAID as of August 2021, in addition to other sequences from Germany [26]. According to the sequences added in the present study, the most recent common ancestor of lineage A.27 dated from April 2020. This was followed by a big dynamic of lineage A.27, especially in February–March 2021, which reached the five continents with multiple introductions in Europe. Since April 2021, variants Alpha and Delta have emerged and displaced lineage A.27 [4]. Currently, lineage A.27 is no longer considered as a VOI, since it seems that it no longer poses significant added risk to global public health. However, its recent detection in Turkey with a slightly modified genetic profile of the isolates suggests the need to further monitor its circulation worldwide.

Despite its high spread in European countries, especially France and Germany, and the outbreak observed in Mayotte (East Africa), the origin of the A.27 lineage was estimated to be West Africa [9,10]; the present study can confirm the West African origin since the earliest cases were described in Niger.

## 5. Conclusions

This work brings additional genomic data and provides an overview on the epidemiology and genetic history of a SARS-CoV-2 lineage that emerged in late 2020 until August 2021. Despite the limitations related to the lack of onsite NGS technologies, the limited resources to test a large number of strains and thus the limited number of strains per country included in the study, the obtained results are not compromised at the African region level, and this work highlights the importance of networking and collaborations in monitoring variants. Although the observed trend worldwide suggests that A.27 has been outcompeted by the B.1.1.7. UK and B.1.617.2 Delta lineages, it may be necessary to continue monitoring the spread of A.27 at the international level, especially since the lineage circulation was relatively recent and the most recent sequences exhibit new mutation profiles that may contribute to its resurgence.

## Figures and Tables

**Table 1:** Demographic and clinical data related to 85 SARS-CoV-2 lineage A.27 sequences reported from Niger, Tunisia, Senegal, Cameroon and Algeria.

Sequence Code	Country	Date of Isolation YYYY-MM-DD	ClinicalForm	Epidemiologicallink	Age (Years)	Gender	GISAID (Accession N°)
hCoV-19/Africa/Algeria/G46955	Algeria	2021-06-14	Unknown	Indigenous	66	M	EPI_ISL_3946135
hCoV-19/Africa/Cameroon/ CPC_21V-8285	Cameroon	2021-02-22	Asymptomatic	Indigenous	26	F	EPI_ISL_8298662
hCoV-19/Africa/Niger/EP40/20	Niger	2020-11-27	Unknown	Indigenous	27	M	EPI_ISL_2281778
hCoV-19/Africa/Niger/Niamey/EP67	Niger	2020-11-27	Unknown	Indigenous	25	M	EPI_ISL_3804025
hCoV-19/Africa/Niger/Niamey/EP75	Niger	2020-11-27	Unknown	Indigenous	28	M	EPI_ISL_3804026
hCoV-19/Africa/Niger/Niamey/CE5523	Niger	2020-11-28	Unknown	Indigenous	19	M	EPI_ISL_2281839
hCoV-19/Africa/Niger/EP132	Niger	2020-11-30	Unknown	Indigenous	29	F	EPI_ISL_2281837
hCoV-19/Africa/Niger/Niamey/CE1574	Niger	2020-11-30	Unknown	Indigenous	51	M	EPI_ISL_2281838
hCoV-19/Africa/Niger/Niamey/CE5715	Niger	2020-11-30	Unknown	Indigenous	30	M	EPI_ISL_2281827
hCoV-19/Africa/Niger/Niamey/17786	Niger	2020-11-31	Asymtomatic	Indigenous	40	M	EPI_ISL_3804022
hCoV-19/Africa/Niger/Agadez/Y/20	Niger	2020-12-04	Unknown	Indigenous		M	EPI_ISL_2281821
hCoV-19/Africa/Niger/EP166	Niger	2020-12-04	Unknown	Indigenous	40	M	EPI_ISL_2281787
hCoV-19/Africa/Niger/EP185	Niger	2020-12-04	Unknown	Indigenous	32	M	EPI_ISL_2281806
hCoV-19/Africa/Niger/Niamey/EP117	Niger	2020-12-04	Unknown	Indigenous	38	M	EPI_ISL_3804030
hCoV-19/Africa/Niger/Niamey/12719	Niger	2020-12-06	Asymptomatic	Indigenous	60	F	EPI_ISL_2281803
hCoV-19/Africa/Niger/Niamey/12734	Niger	2020-12-06	Asymptomatic	Indigenous	37	F	EPI_ISL_2281799
hCoV-19/Africa/Niger/Agadez/76/20	Niger	2020-12-16	Unknown	Indigenous	34	M	**
hCoV-19/Africa/Niger/Niamey/8612	Niger	2020-12-17	Unknown	Indigenous	<1	M	EPI_ISL_3804031
hCoV-19/Africa/Niger/Doutchi/8617	Niger	2020-12-22	Unknown	Indigenous	4	M	EPI_ISL_2281791
hCoV-19/Africa/Niger/Niamey/8637	Niger	2020-12-22	Unknown	Indigenous	8	M	EPI_ISL_3804064
hCoV-19/Africa/Niger/Niamey/16565	Niger	2020-12-24	Asymptomatic	Indigenous	66	F	EPI_ISL_2281798
hCoV-19/Africa/Niger/Niamey/8639	Niger	2020-12-24	Unknown	Indigenous	<1	F	EPI_ISL_3804036
hCoV-19/Africa/Niger/Niamey/16781	Niger	2020-12-25	Unknown	Indigenous	35	M	EPI_ISL_2281834
hCoV-19/Africa/Niger/Agadez/18/20	Niger	2020-12-26	Asymptomatic	Indigenous	23	M	EPI_ISL_2281813
hCoV-19/Africa/Niger/Niamey/16924	Niger	2020-12-26	Asymptomatic	Indigenous	33	F	**
hCoV-19/Africa/Niger/Niamey/16930	Niger	2020-12-26	Asymptomatic	Indigenous	39	M	EPI_ISL_3804057
hCoV-19/Africa/Niger/Niamey/16933	Niger	2020-12-26	Asymptomatic	Indigenous	26	F	EPI_ISL_3804032
hCoV-19/Africa/Niger/Niamey/17065	Niger	2020-12-26	Asymptomatic	Indigenous	10	F	EPI_ISL_3804033
hCoV-19/Africa/Niger/Niamey/17120	Niger	2020-12-26	Asymptomatic	Indigenous	40	M	EPI_ISL_3804022
hCoV-19/Africa/Niger/Niamey/17198	Niger	2020-12-26	Asymptomatic	Indigenous	71	M	EPI_ISL_3804019
hCoV-19/Africa/Niger/Niamey/17204	Niger	2020-12-26	Asymptomatic	Indigenous	4	M	EPI_ISL_3804020
hCoV-19/Africa/Niger/Niamey/17208	Niger	2020-12-26	Asymptomatic	Indigenous	62	M	EPI_ISL_3804021
hCoV-19/Africa/Niger/Niamey/8662	Niger	2020-12-30	Unknown	Indigenous	<1	M	EPI_ISL_2281815
hCoV-19/Africa/Niger/Gaya/8668	Niger	2020-12-31	Unknown	Indigenous	13	M	EPI_ISL_2281777
hCoV-19/Africa/Niger/Niamey/17751	Niger	2020-12-31	Asymtomatic	Indigenous	35	M	EPI_ISL_2281789
hCoV-19/Africa/Niger/Niamey/17753	Niger	2020-12-31	Asymtomatic	Indigenous	27	M	EPI_ISL_2281775
hCoV-19/Africa/Niger/Niamey/17755	Niger	2020-12-31	Asymtomatic	Indigenous	56	M	EPI_ISL_3804058
hCoV-19/Africa/Niger/Niamey/17827	Niger	2021-01-01	Asymtomatic	Indigenous	66	M	EPI_ISL_3804059
hCoV-19/Africa/Niger/Niamey/CE1770	Niger	2021-01-09	Asymtomatic	Indigenous	29	M	EPI_ISL_2281782
hCoV-19/Africa/Niger/Niamey/CNU57	Niger	2021-01-09	Unknown	Indigenous	40	F	EPI_ISL_3804035
hCoV-19/Africa/Niger/CNU180	Niger	2021-01-19	Unknown	Indigenous	47	M	EPI_ISL_3804063
hCoV-19/Africa/Niger/Niamey/19027	Niger	2021-01-19	Mild	Indigenous	39	M	EPI_ISL_3804060
hCoV-19/Africa/Niger/Niamey/19030	Niger	2021-01-19	Mild	Indigenous	47	M	EPI_ISL_3804061
hCoV-19/Africa/Niger/Niamey/19031	Niger	2021-01-19	Mild	Indigenous	64	M	EPI_ISL_3804062
hCoV-19/Africa/Niger/Niamey/CE4805	Niger	2021-01-22	Unknown	Indigenous	42	F	EPI_ISL_3804068
hCoV-19/Africa/Niger/Niamey/CE2069	Niger	2021-02-01	Unknown	Indigenous		M	EPI_ISL_3804066
hCoV-19/Africa/Niger/Niamey/CE729	Niger	2021-02-05	Unknown	Indigenous	63	M	EPI_ISL_2281760
hCoV-19/Africa/Niger/Niamey/19902	Niger	2021-02-17	Asymptomatic	Indigenous	69	M	EPI_ISL_2281759
hCoV-19/Africa/Senegal/156123/	Senegal	2021-01-22	Asymptomatic	Unknown	74	M	xx
hCoV-19/Africa/Senegal/180227	Senegal	2021-02-17	Asymptomatic	Unknown	53	M	xx
hCoV-19/Africa/Senegal/6941172_S6	Senegal	2021-04-12	Asymptomatic	Indigenous	31	F	EPI_ISL_8528025
hCoV-19/Africa/Senegal/6942249_S17	Senegal	2021-04-13	Asymptomatic	Indigenous	42	M	EPI_ISL_8528036
hCoV-19/Africa/Senegal/6944287_S22	Senegal	2021-04-15	Asymptomatic	Indigenous	30	F	EPI_ISL_8528057
hCoV-19/Africa/Senegal/6980248_S62	Senegal	2021-04-19	Asymptomatic	Indigenous	50	F	EPI_ISL_8528097
hCoV-19/Africa/Senegal/6981610_S73	Senegal	2021-04-20	Asymptomatic	Indigenous	55	M	EPI_ISL_8528113
hCoV-19/Africa/Senegal/6981616_S74	Senegal	2021-04-20	Asymptomatic	Indigenous	51	M	EPI_ISL_8528114
hCoV-19/Africa/Senegal/6986192_S79	Senegal	2021-04-21	Asymptomatic	Indigenous	34	M	EPI_ISL_8528124
hCoV-19/Africa/Senegal/6986443_S83	Senegal	2021-04-22	Asymptomatic	Indigenous	42	M	EPI_ISL_8528128
hCoV-19/Africa/Senegal/6986447_S84	Senegal	2021-04-22	Asymptomatic	Indigenous	52	M	EPI_ISL_8528129
hCoV-19/Africa/Senegal/6990626_S101	Senegal	2021-04-25	Asymptomatic	Indigenous	56	M	EPI_ISL_8528160
hCoV-19/Africa/Senegal/6990754_S100	Senegal	2021-04-25	Asymptomatic	Chinese	36	M	EPI_ISL_8528161
hCoV-19/Africa/Senegal/7914449_S260	Senegal	2021-05-20	Asymptomatic	Chinese	30	F	EPI_ISL_8528306
hCoV-19/Africa/Tunisia/Tunis/Q-5509	Tunisia	2021-02-02	Mild	Indigenous	27	F	EPI_ISL_1208398
hCoV-19/Africa/Tunisia/Tunis/Q-5516	Tunisia	2021-02-02	Mild	Indigenous	34	F	EPI_ISL_12316665
hCoV-19/Africa/Tunisia/Tunis/Q-6575	Tunisia	2021-02-07	Mild	Indigenous		M	EPI_ISL_1207287
hCoV-19/Africa/Tunisia/Tunis/Q-6590	Tunisia	2021-02-07	Mild	Indigenous	17	M	EPI_ISL_2035942
hCoV-19/Africa/Tunisia/Tunis/Q-9052	Tunisia	2021-02-23	Mild	Indigenous	78	M	EPI_ISL_12316666
hCoV-19/Africa/Tunisia/Tunis/Q-9058	Tunisia	2021-02-23	Mild	Indigenous	79	M	**
hCoV-19/Africa/Tunisia/Tunis/Q-9081	Tunisia	2021-02-23	Mild	Indigenous	31	M	EPI_ISL_12316667
hCoV-19/Africa/Tunisia/Tunis/Q-9082	Tunisia	2021-02-23	Mild	Indigenous	85	F	EPI_ISL_12316668
hCoV-19/Africa/Tunisia/Tunis/U-4071	Tunisia	2021-02-24	Asymptomatic	Cote d’Ivoire	36	M	xx
hCoV-19/Africa/Tunisia/Tunis/Q-9407	Tunisia	2021-02-25	Mild	Indigenous	32	M	EPI_ISL_10101339
hCoV-19/Africa/Tunisia/Tunis/Q-9581	Tunisia	2021-02-25	Mild	Indigenous	80	M	EPI_ISL_12316669
hCoV-19/Africa/Tunisia/Ariana/Q-9623	Tunisia	2021-02-25	Mild	Indigenous	25	F	EPI_ISL_10101331
hCoV-19/Africa/Tunisia/Siliana/Q-9993	Tunisia	2021-03-01	Severe	Indigenous	33	M	EPI_ISL_12316670
hCoV-19/Africa/Tunisia/Siliana/B-0386	Tunisia	2021-03-03	Mild	Indigenous	65	F	xx
hCoV-19/Africa/Tunisia/Tunis/B-0396	Tunisia	2021-03-03	Mild	Indigenous	67	F	EPI_ISL_2035946
hCoV-19/Africa/Tunisia/Tunis/B-1388	Tunisia	2021-03-09	Mild	Indigenous	30	F	xx
hCoV-19/Africa/Tunisia/Manouba/S-0159	Tunisia	2021-03-16	Mild	Indigenous	34	F	EPI_ISL_10101347
hCoV-19/Africa/Tunisia/Mednine/S-0418	Tunisia	2021-04-20	Mild	Libya	54	M	xx
hCoV-19/Africa/Tunisia/Ben Arous/C-4468	Tunisia	2021-04-21	Mild	Indigenous	20	F	EPI_ISL_10141399
hCoV-19/Africa/Tunisia/Ben Arous/C-4469	Tunisia	2021-04-21	Mild	Indigenous	14	M	EPI_ISL_10141512
hCoV-19/Africa/Tunisia/Tunis/C-5524	Tunisia	2021-04-23	Severe	Indigenous	72	M	EPI_ISL_10141507
hCoV-19/Africa/Tunisia/Manouba/S-0042	Tunisia	2021-03-16	Mild	Indigenous	68	F	EPI_ISL_12280586
hCoV-19/Africa/Tunisia/Manouba/S-0043	Tunisia	2021-03-16	Mild	Indigenous	44	M	EPI_ISL_10101222

The earliest strains of SARS-CoV-2 lineage A.27 detected. ** Not published in GISAID because of the high number of ambiguous nucleotides. The sequence is classified as A.27 based on specific mutations in the S gene.

## Data Availability

Not applicable.

## Supplementary Materials

The following supporting information can be downloaded at: https://www.mdpi.com/article/10.3390/v14051007/s1, Supplementary Material S1: The Maximum Clade Credibility (MCC) Tree generated by BEAST. Supplementary Material S2: Main amino acid (aa) substitutions that characterize the A.27 lineage.
