# Supplementary material for: SARS-CoV-2 Lineage A.27: New Data from African Countries and Dynamics in the Context of the COVID-19 Pandemic
Source: Viruses. 2022 May 9;14(5):1007. doi: 10.3390/v14051007 (PMC9144831; doi:10.3390/v14051007)
Supplement: Supplementary file 1 [file viruses-14-01007-s001.zip › Legend Supplementary Material S1.pdf]

The Maximum Clade Credibility (MCC) Tree generated by BEAST.
TreeAnnotator was used to summarize the 1000 tree data produced by BEAST onto a single tree called Maximum Clade Credibility Tree (MCC). The burnin (as states) was fixed to 100 equivalent to 10% of the total number of trees generated by BEAST. The posterior probability limit was fixed to 0.7. Posterior summaries were only calculated for nodes in the target tree that have a posterior probability greater than the specified limit. Nodes with very little support were not considered.
