# Supplementary material for: SARS-CoV-2 Lineage A.27: New Data from African Countries and Dynamics in the Context of the COVID-19 Pandemic
Source: Viruses. 2022 May 9;14(5):1007. doi: 10.3390/v14051007 (PMC9144831; doi:10.3390/v14051007)
Supplement: Supplementary file 1 [file viruses-14-01007-s001.zip › Supplementary material S3_gisaid_hcov-19_acknowledgement_table_2022_03_08_09.pdf]

We gratefully acknowledge the following Authors from the Originating laboratories responsible for obtaining the specimens, as well as the Submitting laboratories where the genome data were generated and shared via GISAID, on which this research is based.

All Submitters of data may be contacted directly via www.gisaid.org

Authors are sorted alphabetically.

Acknowledgement EPI_SET Identifier: EPI_SET_20220308za

Accession ID	Originating Laboratory	Submitting Laboratory	Authors
EPI_ISL_4537460	APHP Bichat-Claude Bernard	APHP Bichat-Claude Bernard	Benoit Visseaux; Samuel Lebourgeois
EPI_ISL_1155709, EPI_ISL_1155711, EPI_ISL_1155713	AX BIO OCEAN	CNR Virus des Infections Respiratoires - France SUD	Antonin Bal; Bruno Lina; Bruno Simon; Gregory Destras; Gwendolyne Burfin; Hadrien Regue; Laurence Josset; Martine Valette; Quentin Semanas
EPI_ISL_1290839, EPI_ISL_1290840	AXBIO	CNR Virus des Infections Respiratoires - France SUD	Antonin Bal; Bruno Lina; Bruno Simon; Gregory Destras; Gwendolyne Burfin; Hadrien Regue; Laurence Josset; Martine Valette; Quentin Semanas
EPI_ISL_1754503, EPI_ISL_2259273, EPI_ISL_2259279	BIOMNIS EUROFINS IVRY	Department of Virology, Henri Mondor University Hospital, Assistance Publique Hôpitaux de Paris, Université Paris-Est Créteil, INSERM U955	Alexandre Soulier; Christophe Rodriguez; Elisabeth Trawinski; Guillaume Gricourt; Jean-Michel Pawlotsky; Melissa N'Debi; Slim Fourati; Vanessa Demontant
EPI_ISL_1239445, EPI_ISL_1253541, EPI_ISL_1253542, EPI_ISL_1253544, EPI_ISL_1253549, EPI_ISL_1253550, EPI_ISL_1253551, EPI_ISL_1290837, EPI_ISL_1290841, EPI_ISL_1312377, EPI_ISL_1312378			
see above	BIOMNIS LYON	CNR Virus des Infections Respiratoires - France SUD	Antonin Bal; Bruno Lina; Bruno Simon; Gregory Destras; Gwendolyne Burfin; Hadrien Regue; Laurence Josset; Martine Valette; Quentin Semanas
EPI_ISL_1312372, EPI_ISL_1312373, EPI_ISL_1312374, EPI_ISL_1312375	BIOMNIS PARIS	CNR Virus des Infections Respiratoires - France SUD	Antonin Bal; Bruno Lina; Bruno Simon; Gregory Destras; Gwendolyne Burfin; Hadrien Regue; Laurence Josset; Martine Valette; Quentin Semanas
EPI_ISL_1253568, EPI_ISL_1253569, EPI_ISL_1253570, EPI_ISL_1253571, EPI_ISL_1253572, EPI_ISL_1253573, EPI_ISL_1253577, EPI_ISL_1253578			
see above	BOURG LES VALENCE	CNR Virus des Infections Respiratoires - France SUD	Antonin Bal; Bruno Lina; Bruno Simon; Gregory Destras; Gwendolyne Burfin; Hadrien Regue; Laurence Josset; Martine Valette; Quentin Semanas
EPI_ISL_1695975, EPI_ISL_1696011, EPI_ISL_1696013, EPI_ISL_2179502	BROUSSAIS	Department of Virology, Henri Mondor University Hospital, Assistance Publique Hôpitaux de Paris, Université Paris-Est Créteil, INSERM U955	Alexandre Soulier; Christophe Rodriguez; Elisabeth Trawinski; Guillaume Gricourt; Jean-Michel Pawlotsky; Melissa N'Debi; Slim Fourati; Vanessa Demontant
EPI_ISL_1707275, EPI_ISL_1707792	Biogroup Bio Lam-LCD Saint-Denis	Department of Virology, Henri Mondor University Hospital, Assistance Publique Hôpitaux de Paris, Université Paris-Est Créteil, INSERM U955	Alexandre Soulier; Christophe Rodriguez; Elisabeth Trawinski; Guillaume Gricourt; Jean-Michel Pawlotsky; Melissa N'Debi; Slim Fourati; Vanessa Demontant
EPI_ISL_1749368	Biology Lab, HIA BEGIN	IRBA, 2MI	CHAPUS C.; DEPEILLE A.; GORGE O.; GRANDPERRET V.; JARJAVAL F.; MERENS-GONTIER A.; NOLENT F.; SARILAR V.; VERGUET N.
EPI_ISL_1706829, EPI_ISL_1706830, EPI_ISL_1706832, EPI_ISL_1706833, EPI_ISL_1706835	C.H.C.B KERIO	Department of Virology, Henri Mondor University Hospital, Assistance Publique Hôpitaux de Paris, Université Paris-Est Créteil, INSERM U955	Alexandre Soulier; Christophe Rodriguez; Elisabeth Trawinski; Guillaume Gricourt; Jean-Michel Pawlotsky; Melissa N'Debi; Slim Fourati; Vanessa Demontant
EPI_ISL_1287759	CDP HUSSEL VIENNE	CNR Virus des Infections Respiratoires - France SUD	Antonin Bal; Bruno Lina; Bruno Simon; Gregory Destras; Gwendolyne Burfin; Hadrien Regue; Laurence Josset; Martine Valette; Quentin Semanas
EPI_ISL_1287752	CERBALLIANCE PACA	CNR Virus des Infections Respiratoires - France SUD	Antonin Bal; Bruno Lina; Bruno Simon; Gregory Destras; Gwendolyne Burfin; Hadrien Regue; Laurence Josset; Martine Valette; Quentin Semanas
EPI_ISL_1313012, EPI_ISL_1313014	CERBALLIANCE RHONE ALPES	CNR Virus des Infections Respiratoires - France SUD	Antonin Bal; Bruno Lina; Bruno Simon; Gregory Destras; Gwendolyne Burfin; Hadrien Regue; Laurence Josset; Martine Valette; Quentin Semanas
EPI_ISL_1910917	CH Bethune	CHU Lille	AIT YAHYA Emilie; ALIDJINOU Enagnon Kazali; BOCKET Laurence; CREPIN Michel; DEMAY Christophe; ENGELMANN Ilka; GEFFROY Sandrine; GUIGON Aurélie; LAMBERT Valérie; LAZREK Mouna; NOBILLIAUX Florian; PREVOST Brigitte; THUILLIER Caroline; TINEZ Claire
EPI_ISL_1671818	CH Bethune	CHU Lille - Laboratoire de Virologie	AIT YAHYA Emilie; ALIDJINOU Enagnon Kazali; BOCKET Laurence; CREPIN Michel; DEMAY Christophe; ENGELMANN Ilka; GEFFROY Sandrine; GUIGON Aurélie; LAMBERT Valérie; LAZREK Mouna; NOBILLIAUX Florian; PREVOST Brigitte; THUILLIER Caroline; TINEZ Claire
EPI_ISL_4646396	CH CAHORS	CHU Purpan - Laboratoire de Virologie - Institut Fédératif de Biologie	Bulach T.; Donnadieu C.; Izopet J.; Latour J.; Milhes M.; Nicot F.; Ranger N.; Salin G.; Tremeaux P.
EPI_ISL_1448016	CH MAUBEUGE	CHU Lille - Laboratoire de Virologie	AIT YAHYA Emilie; ALIDJINOU Enagnon Kazali; BOCKET Laurence; CREPIN Michel; DEMAY Christophe; ENGELMANN Ilka; GEFFROY Sandrine; GUIGON Aurélie; LAMBERT Valérie; LAZREK Mouna; NOBILLIAUX Florian; PREVOST Brigitte; TCHANTCHOU NJOSSE YANICK; THUILLIER Caroline; TINEZ Claire
EPI_ISL_1707498	CH VALENCE	CNR Virus des Infections Respiratoires - France SUD	Antonin Bal; Bruno Lina; Bruno Simon; Gregory Destras; Gwendolyne Burfin; Hadrien Regue; Laurence Josset; Martine Valette; Quentin Semanas
EPI_ISL_1672661	CH.INTERCOMMUNAL DE CRETEIL	Department of Virology, Henri Mondor University Hospital, Assistance Publique Hôpitaux de Paris, Université Paris-Est Créteil, INSERM U955	Alexandre Soulier; Christophe Rodriguez; Elisabeth Trawinski; Guillaume Gricourt; Jean-Michel Pawlotsky; Melissa N'Debi; Slim Fourati; Vanessa Demontant
EPI_ISL_2011683	CHU Angoulême	CHU Poitiers	Agnes BEBY-DEFAUX; Birama N'DIAYE; Caroline MICHAUD; Manon PRAT; Maxime PICHON; Nicolas LEVEQUE
EPI_ISL_6157234	CHU Pontchaillou	CHU Pontchaillou	DE TAYRAC Marie; DENOUAL Florent; ETCHEVERRY Amandine; FEBREAU Christine; GALIBERT Marie Dominique; GROLHIER Claire; JAGLINE Steven; PRONIER Charlotte; QUENET Benjamin; SASSI Mohamed; THIBAULT Vincent
EPI_ISL_1425079, EPI_ISL_1448425, EPI_ISL_4647979	CHU Purpan - Laboratoire de Virologie - Institut Fédératif de Biologie	CHU Purpan - Laboratoire de Virologie - Institut Fédératif de Biologie	Agnès Harter; Bulach T.; Donnadieu C.; Izopet J.; Jacques Izopet; Justine Latour; Latour J.; Martine Dubois; Milhes M.; Nicolas Jeanne; Nicot F.; Noémie Ranger; Pauline Boyer; Pauline Tremeaux; Ranger N.; Romain Carcenac; Salin G.; Tremeaux P.
EPI_ISL_1290836	CHU Rennes	CNR Virus des Infections Respiratoires - France SUD	Antonin Bal; Bruno Lina; Bruno Simon; Gregory Destras; Gwendolyne Burfin; Hadrien Regue; Laurence Josset; Martine Valette; Quentin Semanas
EPI_ISL_1313709, EPI_ISL_1707485	CHU ST ETIENNE HOPITAL NORD	CNR Virus des Infections Respiratoires - France SUD	Antonin Bal; Bruno Lina; Bruno Simon; Gregory Destras; Gwendolyne Burfin; Hadrien Regue; Laurence Josset; Martine Valette; Quentin Semanas
EPI_ISL_1239446, EPI_ISL_1253567, EPI_ISL_1312825, EPI_ISL_1313667, EPI_ISL_1313693, EPI_ISL_1314016, EPI_ISL_1707523, EPI_ISL_1707526, EPI_ISL_1707528, EPI_ISL_1707536, EPI_ISL_1707537			
see above	CNR Virus des Infections Respiratoires - France SUD	CNR Virus des Infections Respiratoires - France SUD	Antonin Bal; Bruno Lina; Bruno Simon; Gregory Destras; Gwendolyne Burfin; Hadrien Regue; Laurence Josset; Martine Valette; Quentin Semanas
EPI_ISL_1483780	Centre De Prelevement COVID RIOM	CHU Clermont-Ferrand, service de virologie	Bisseux Maxime; Combes Patricia; Henquell Cécile; Mirand Audrey
EPI_ISL_1706459	Centre Hospitalier Eure Seine	Centre Hospitalier Universitaire de Rouen Laboratoire de Virologie	Alice Moisan; Fabienne De Oliveira; Marie Leoz
EPI_ISL_1483774, EPI_ISL_1483776, EPI_ISL_1483786, EPI_ISL_1490236	Centre Hospitalier Universitaire Clermont-Ferrand	CHU Clermont-Ferrand, service de virologie	Bisseux Maxime; Combes Patricia; Henquell Cécile; Mirand Audrey
EPI_ISL_4646385	Centre Hospitalier de Lavaur	CHU Purpan - Laboratoire de Virologie - Institut Fédératif de Biologie	Bulach T.; Donnadieu C.; Izopet J.; Latour J.; Milhes M.; Nicot F.; Ranger N.; Salin G.; Tremeaux P.
EPI_ISL_1312896	DYOMEDEA LYON	CNR Virus des Infections Respiratoires - France SUD	Antonin Bal; Bruno Lina; Bruno Simon; Gregory Destras; Gwendolyne Burfin; Hadrien Regue; Laurence Josset; Martine Valette; Quentin Semanas
EPI_ISL_1457768	DYOMEDEA SAUVEGARDE LYON	CNR Virus des Infections Respiratoires - France SUD	Antonin Bal; Bruno Lina; Bruno Simon; Gregory Destras; Gwendolyne Burfin; Hadrien Regue; Laurence Josset; Martine Valette; Quentin Semanas
EPI_ISL_1696047	E.F.S B.F.C JEAN MINJOZ	Department of Virology, Henri Mondor University Hospital, Assistance Publique Hôpitaux de Paris, Université Paris-Est Créteil, INSERM U955	Alexandre Soulier; Christophe Rodriguez; Elisabeth Trawinski; Guillaume Gricourt; Jean-Michel Pawlotsky; Melissa N'Debi; Slim Fourati; Vanessa Demontant
EPI_ISL_1110194, EPI_ISL_1110195, EPI_ISL_1110196, EPI_ISL_1110197, EPI_ISL_1110198, EPI_ISL_1672700, EPI_ISL_1672744, EPI_ISL_1672745, EPI_ISL_1672746, EPI_ISL_1672747, EPI_ISL_1672748, EPI_ISL_1672749, EPI_ISL_1706658, EPI_ISL_1706661			
see above	G.H.E.F.Grand Hôpital EST Francilien	Department of Virology, Henri Mondor University Hospital, Assistance Publique Hôpitaux de Paris, Université Paris-Est Créteil, INSERM U955	Alexandre Soulier; Christophe Rodriguez; Elisabeth Trawinski; Guillaume Gricourt; Jean-Michel Pawlotsky; Melissa N'Debi; Slim Fourati; Vanessa Demontant
EPI_ISL_1672460, EPI_ISL_1672547, EPI_ISL_1755046, EPI_ISL_2179448, EPI_ISL_2259268	GH A.CHENEVIER-H.MONDOR	Department of Virology, Henri Mondor University Hospital, Assistance Publique Hôpitaux de Paris, Université Paris-Est Créteil, INSERM U955	Alexandre Soulier; Christophe Rodriguez; Elisabeth Trawinski; Guillaume Gricourt; Jean-Michel Pawlotsky; Melissa N'Debi; Slim Fourati; Vanessa Demontant
EPI_ISL_1672499	GH JOFFRE DUPUYTREN	Department of Virology, Henri Mondor University Hospital, Assistance Publique Hôpitaux de Paris, Université Paris-Est Créteil, INSERM U955	Alexandre Soulier; Christophe Rodriguez; Elisabeth Trawinski; Guillaume Gricourt; Jean-Michel Pawlotsky; Melissa N'Debi; Slim Fourati; Vanessa Demontant
EPI_ISL_2179373, EPI_ISL_2179568, EPI_ISL_2179569, EPI_ISL_2179576	GH de l'Est Francilien	Department of Virology, Henri Mondor University Hospital, Assistance Publique Hôpitaux de Paris, Université Paris-Est Créteil, INSERM U955	Alexandre Soulier; Christophe Rodriguez; Elisabeth Trawinski; Guillaume Gricourt; Jean-Michel Pawlotsky; Melissa N'Debi; Slim Fourati; Vanessa Demontant
EPI_ISL_1696300, EPI_ISL_1696301	Groupe LCD	Department of Virology, Henri Mondor University Hospital, Assistance Publique Hôpitaux de Paris, Université Paris-Est Créteil, INSERM U955	Alexandre Soulier; Christophe Rodriguez; Elisabeth Trawinski; Guillaume Gricourt; Jean-Michel Pawlotsky; Melissa N'Debi; Slim Fourati; Vanessa Demontant

EPI_ISL_1731504	HOPITAL DU MONT DORE	CHU Clermont-Ferrand, service de virologie	Bisseux Maxime; Combes Patricia; Henquell Cécile; Mirand Audrey
EPI_ISL_1239447	HOPITAL MARIN HENDAYE	CNR Virus des Infections Respiratoires - France SUD	Antonin Bal; Bruno Lina; Bruno Simon; Gregory Destras; Gwendolyne Burfin; Hadrien Regue; Laurence Josset; Martine Valette; Quentin Semanas
EPI_ISL_1111061, EPI_ISL_1118883, EPI_ISL_1118891, EPI_ISL_1118898, EPI_ISL_1118899, EPI_ISL_1239371, EPI_ISL_1259300, EPI_ISL_1259305, EPI_ISL_1336334, EPI_ISL_2178465, EPI_ISL_2363514			
see above	Hopital	National Reference Center for Viruses of Respiratory Infections, Institut Pasteur, Paris	Angela Brisebarre; Camille Capel; Christophe Malabat; Corinne Maufrais; Damien Mornico; Ducancelle Alexandra; Ducancelle Alexandra; Etienne Simon-Lorière; Frédéric Lemoine; Gastli Nabil; Lagathu GisèLe; Louise Lefrançois; Luizy Nelly; Marion Barbet; Martres Pascale; Maud Vanpeene; Méline Bizard; Scanvic AgnèS; Sylvie Behillil; Sylvie van der Werf; Vincent Enouf
EPI_ISL_1381819, EPI_ISL_1381820, EPI_ISL_1381824, EPI_ISL_1381825, EPI_ISL_1381826, EPI_ISL_1381827, EPI_ISL_1381831, EPI_ISL_1443884, EPI_ISL_1517032, EPI_ISL_1582704, EPI_ISL_1582708, EPI_ISL_1583043, EPI_ISL_2178413, EPI_ISL_2178501			
see above	Hospital	National Reference Center for Viruses of Respiratory Infections, Institut Pasteur, Paris	Amaury Vaysse; Angela Brisebarre; Bressollette CéLine; Camille Capel; Christophe Malabat; CléMence Guillaume; Corinne Maufrais; CéLine Bressollette; Etienne Simon-Lorière; Frédéric Lemoine; Gastli Nabil; JéRome Guinard; Louise Lefrançois; LéA Pilorge; Marion Barbet; Maud Vanpeene; Méline Bizard; Pierre Lechat; Pilorge LéA; Sylvie Behillil; Sylvie Van der Werf; Sylvie van der Werf; Thibault Guinoiseau; Vincent Enouf
EPI_ISL_1110181, EPI_ISL_1110207, EPI_ISL_1672676, EPI_ISL_1672679, EPI_ISL_1672704	Hôpital Avicenne	Department of Virology, Henri Mondor University Hospital, Assistance Publique Hôpitaux de Paris, Université Paris-Est Créteil, INSERM U955	Alexandre Soulier; Christophe Rodriguez; Elisabeth Trawinski; Guillaume Gricourt; Jean-Michel Pawlotsky; Melissa N'Debi; Slim Fourati; Vanessa Demontant
EPI_ISL_2467555, EPI_ISL_2467575, EPI_ISL_2467672	Hôpital Bichat Claude Bernard, Laboratoire de Virologie	IAME UMR1137 Inserm, Université de Paris, Hôpital Bichat	Alexandre Storto; Amélie Recoing; Antoine Bridier-Nahmias; Benoit Visseaux; Charlotte Charpentier; Diane Descamps; Gilles Collin; Lena Daniel; Mélanie Bertine; Nadhira Houhou-Fidouh; Quentin Le Hingrat; Siham Hamri
EPI_ISL_1085610, EPI_ISL_1085617, EPI_ISL_1088552, EPI_ISL_1088554, EPI_ISL_1088565, EPI_ISL_1088566, EPI_ISL_1110156, EPI_ISL_1110157, EPI_ISL_1110173, EPI_ISL_1110178, EPI_ISL_1110203			
see above	Hôpital Henri Mondor	Department of Virology, Henri Mondor University Hospital, Assistance Publique Hôpitaux de Paris, Université Paris-Est Créteil, INSERM U955	Alexandre Soulier; Christophe Rodriguez; Elisabeth Trawinski; Guillaume Gricourt; Jean-Michel Pawlotsky; Melissa N'Debi; Slim Fourati; Vanessa Demontant
EPI_ISL_1672723, EPI_ISL_1672794	Hôpital Paul Brousse	Department of Virology, Henri Mondor University Hospital, Assistance Publique Hôpitaux de Paris, Université Paris-Est Créteil, INSERM U955	Alexandre Soulier; Christophe Rodriguez; Elisabeth Trawinski; Guillaume Gricourt; Jean-Michel Pawlotsky; Melissa N'Debi; Slim Fourati; Vanessa Demontant
EPI_ISL_1110170, EPI_ISL_1110210	Hôpital Pitié-Salpêtrière	Department of Virology, Henri Mondor University Hospital, Assistance Publique Hôpitaux de Paris, Université Paris-Est Créteil, INSERM U955	Alexandre Soulier; Christophe Rodriguez; Elisabeth Trawinski; Guillaume Gricourt; Jean-Michel Pawlotsky; Melissa N'Debi; Slim Fourati; Vanessa Demontant
EPI_ISL_1672452, EPI_ISL_1672453	Hôpitaux Universitaires de Strasbourg NHC	Department of Virology, Henri Mondor University Hospital, Assistance Publique Hôpitaux de Paris, Université Paris-Est Créteil, INSERM U955	Alexandre Soulier; Christophe Rodriguez; Elisabeth Trawinski; Guillaume Gricourt; Jean-Michel Pawlotsky; Melissa N'Debi; Slim Fourati; Vanessa Demontant
EPI_ISL_1097023, EPI_ISL_5032150	IHU Mediterranee Infection	IHU Mediterranee Infection	Philippe Colson; Philippe Colson et al.
EPI_ISL_6101653	LABO BIO DOC	CHU Purpan - Laboratoire de Virologie - Institut Fédératif de Biologie	Bulach T.; Donnadieu C.; Izopet J.; Latour J.; Milhes M.; Nicot F.; Ranger N.; Salin G.; Tremeaux P.
EPI_ISL_1313043	LABORATOIRE ALPIGENE	CNR Virus des Infections Respiratoires - France SUD	Antonin Bal; Bruno Lina; Bruno Simon; Gregory Destras; Gwendolyne Burfin; Hadrien Regue; Laurence Josset; Martine Valette; Quentin Semanas
EPI_ISL_4646397	LABORATOIRE BIO3	CHU Purpan - Laboratoire de Virologie - Institut Fédératif de Biologie	Bulach T.; Donnadieu C.; Izopet J.; Latour J.; Milhes M.; Nicot F.; Ranger N.; Salin G.; Tremeaux P.
EPI_ISL_1403498	LBA Casteljaloux	Cerba Lab	Benazra M; Haïm-Boukobza S; Lecorche E; Olivi M; Roquebert B; Trombert-Paolantoni S; Zimmer S
EPI_ISL_1253552, EPI_ISL_1253553, EPI_ISL_1253554, EPI_ISL_1287741, EPI_ISL_1287742, EPI_ISL_1287743, EPI_ISL_1287744, EPI_ISL_1287747, EPI_ISL_1287749, EPI_ISL_1287750, EPI_ISL_1287751, EPI_ISL_1312994, EPI_ISL_1313000, EPI_ISL_1313001, EPI_ISL_1314035, EPI_ISL_1314039, EPI_ISL_1935386			
see above	LBM UNIBIO VALENTIN	CNR Virus des Infections Respiratoires - France SUD	Antonin Bal; Bruno Lina; Bruno Simon; Gregory Destras; Gwendolyne Burfin; Hadrien Regue; Laurence Josset; Martine Valette; Quentin Semanas
EPI_ISL_1313015	LX BIO	CNR Virus des Infections Respiratoires - France SUD	Antonin Bal; Bruno Lina; Bruno Simon; Gregory Destras; Gwendolyne Burfin; Hadrien Regue; Laurence Josset; Martine Valette; Quentin Semanas
EPI_ISL_1118879, EPI_ISL_1118880, EPI_ISL_1118881, EPI_ISL_1118882, EPI_ISL_1118902, EPI_ISL_1118903, EPI_ISL_1118904, EPI_ISL_1118905, EPI_ISL_1118906, EPI_ISL_1118907, EPI_ISL_1118908, EPI_ISL_1118909, EPI_ISL_1259298, EPI_ISL_1259301, EPI_ISL_1259302, EPI_ISL_1259303, EPI_ISL_1336331, EPI_ISL_1336332, EPI_ISL_1336333, EPI_ISL_1443910, EPI_ISL_1623013, EPI_ISL_1739306, EPI_ISL_1739316, EPI_ISL_2178452, EPI_ISL_2178453, EPI_ISL_2178454, EPI_ISL_2178455, EPI_ISL_2178456, EPI_ISL_2178457, EPI_ISL_2178458, EPI_ISL_2178459, EPI_ISL_2178460, EPI_ISL_2178461, EPI_ISL_2178462, EPI_ISL_2178463, EPI_ISL_2259091, EPI_ISL_2363516, EPI_ISL_4211287			
see above	Labo Analyses Med	National Reference Center for Viruses of Respiratory Infections, Institut Pasteur, Paris	Amaury Vaysse; Angela Brisebarre; Bonnet Cyrile; Camille Capel; Christophe Malabat; Coignard Catherine; Corinne Maufrais; Damien Mornico; Desjardins Nicolas; Ducancelle Alexandra; Etienne Simon-Lorière; Fabienne Artur; Frédéric Lemoine; Holstein Anne; Hub de Bioinformatique et Biostatistiques; King Lisa; Le Berre David; Le Vicky; Louise Lefrançois; Mallet C; Marion Barbet; Maud Vanpeene; Melanie Caron; Méline Bizard; OphéLie Said-Delattre; Pierre Lechat; Sylvie Behillil; Sylvie Van der Werf; Sylvie van der Werf; Vincent Enouf
EPI_ISL_1754272	Laboratoire CBM 25 TERRE ROUGE	Department of Virology, Henri Mondor University Hospital, Assistance Publique Hôpitaux de Paris, Université Paris-Est Créteil, INSERM U955	Alexandre Soulier; Christophe Rodriguez; Elisabeth Trawinski; Guillaume Gricourt; Jean-Michel Pawlotsky; Melissa N'Debi; Slim Fourati; Vanessa Demontant
EPI_ISL_1201045, EPI_ISL_1201046, EPI_ISL_7807004	MEPHI, Aix Marseille University	MEPHI, Aix Marseille University	Anthony LEVASSEUR
EPI_ISL_1587554, EPI_ISL_1587569, EPI_ISL_1587570	NOVABIO BERGERAC	CNR Virus des Infections Respiratoires - France SUD	Antonin Bal; Bruno Lina; Bruno Simon; Gregory Destras; Gwendolyne Burfin; Hadrien Regue; Laurence Josset; Martine Valette; Quentin Semanas
EPI_ISL_1707512, EPI_ISL_1707513, EPI_ISL_1707514, EPI_ISL_1707515, EPI_ISL_1707517	NOVABIO DORDOGNE	CNR Virus des Infections Respiratoires - France SUD	Antonin Bal; Bruno Lina; Bruno Simon; Gregory Destras; Gwendolyne Burfin; Hadrien Regue; Laurence Josset; Martine Valette; Quentin Semanas
EPI_ISL_1253574, EPI_ISL_1253575, EPI_ISL_1253576	NOVELAB Ingels Vignon	CNR Virus des Infections Respiratoires - France SUD	Antonin Bal; Bruno Lina; Bruno Simon; Gregory Destras; Gwendolyne Burfin; Hadrien Regue; Laurence Josset; Martine Valette; Quentin Semanas
EPI_ISL_934974	Novabio	CNR Virus des Infections Respiratoires - France SUD	Antonin Bal; Bruno Lina; Gregory Destras; Gwendolyne Burfin; Hadrien Règue; Laurence Josset; Martine Valette; Quentin Semanas
EPI_ISL_6157233	Pontivy SIDEP	CHU Pontchaillou	DE TAYRAC Marie; DENOUAL Florent; ETCHEVERRY Amandine; FEBREAU Christine; GALIBERT Marie Dominique; GROLHIER Claire; JAGLINE Steven; PRONIER Charlotte; QUENET Benjamin; SASSI Mohamed; THIBAULT Vincent
EPI_ISL_6101630, EPI_ISL_6101631	SELAS CBM MURET CUGNOT	CHU Purpan - Laboratoire de Virologie - Institut Fédératif de Biologie	Bulach T.; Donnadieu C.; Izopet J.; Latour J.; Milhes M.; Nicot F.; Ranger N.; Salin G.; Tremeaux P.
EPI_ISL_1787536	Synlab Haut de France	UMR 8199/1283 EGID	Derhourhi Mehdi
EPI_ISL_2131453	UMR190-Unité des virus emergents	UMR190-Unité des virus emergents	cecile Baronti
EPI_ISL_1290838, EPI_ISL_1312895	UNIBIO	CNR Virus des Infections Respiratoires - France SUD	Antonin Bal; Bruno Lina; Bruno Simon; Gregory Destras; Gwendolyne Burfin; Hadrien Regue; Laurence Josset; Martine Valette; Quentin Semanas
EPI_ISL_1707511	UNIBIO ROMANS GAMBETTA	CNR Virus des Infections Respiratoires - France SUD	Antonin Bal; Bruno Lina; Bruno Simon; Gregory Destras; Gwendolyne Burfin; Hadrien Regue; Laurence Josset; Martine Valette; Quentin Semanas
EPI_ISL_2094433, EPI_ISL_2491373	cerballiance-IDF	Cerba lab	Aude Lessenne; Bénédicte Roquebert; Emmanuel Lecorche; Kader Merah; Laura Verdurme; Patrice Herisson; Sabine Trombert-Paolantoni; Stéphanie Haïm-Boukobza; Thierry Collin
EPI_ISL_1336339, EPI_ISL_1336340	hopital	National Reference Center for Viruses of Respiratory Infections, Institut Pasteur, Paris	Angela Brisebarre; Camille Capel; Etienne Simon-Lorière; Gastli Nabil; Marion Barbet; Maud Vanpeene; Méline Bizard; Sylvie Behillil; Sylvie van der Werf; Vincent Enouf
EPI_ISL_1696154, EPI_ISL_1696182	laboratoire Belle Epine	Department of Virology, Henri Mondor University Hospital, Assistance Publique Hôpitaux de Paris, Université Paris-Est Créteil, INSERM U955	Alexandre Soulier; Christophe Rodriguez; Elisabeth Trawinski; Guillaume Gricourt; Jean-Michel Pawlotsky; Melissa N'Debi; Slim Fourati; Vanessa Demontant
