# Supplementary figures and images for: SARS-CoV-2 Lineage A.27: New Data from African Countries and Dynamics in the Context of the COVID-19 Pandemic
Source: Viruses. 2022 May 9;14(5):1007. doi: 10.3390/v14051007 (PMC9144831; doi:10.3390/v14051007)

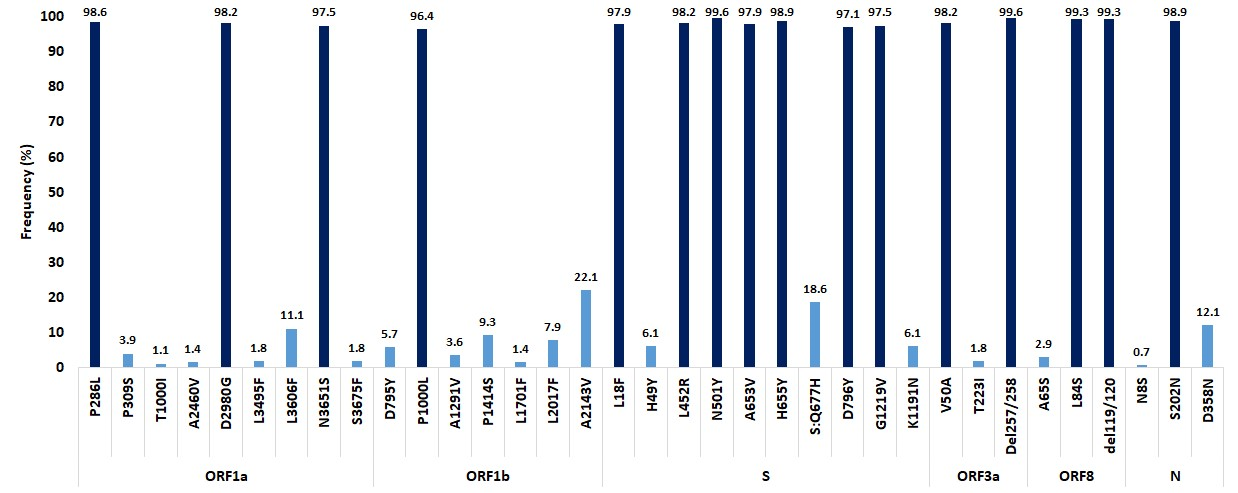

Supplement: Supplementary file 1 [file viruses-14-01007-s001.zip › Supplementary Material S2.tif]
